# Association between the triglyceride-glucose-waist-to-height ratio and cardiovascular disease in Chinese adults with sarcopenia or probable sarcopenia

**DOI:** 10.3389/fendo.2025.1686885

**Published:** 2025-10-13

**Authors:** Wei Huang, Jin Wu, Zhimei Shen, Dasheng Wang, Xu Wang

**Affiliations:** ^1^ Health Management Center, Northern Jiangsu People’s Hospital Affiliated to Yangzhou University, Yangzhou, China; ^2^ Northern Jiangsu People’s Hospital, Yangzhou, China; ^3^ Department of Emergency Medicine, Northern Jiangsu People’s Hospital Affiliated to Yangzhou University, Yangzhou, China

**Keywords:** TyG, WHtR, sarcopenia, cardiovascular disease, stroke

## Abstract

**Introduction:**

Sarcopenia, an age-related syndrome characterized by decreased muscle mass and performance, has been increasingly linked to high cardiovascular disease (CVD) risk. In this study, sarcopenia and probable sarcopenia were diagnosed according to the Asian Working Group for Sarcopenia (AWGS) 2019 criteria. However, specific biomarkers underlying this association, such as the triglyceride-glucose-waist-to-height ratio (TyG-WHtR), remain unclear.

**Methods:**

A cohort of 2,521 adults ≥45 years with sarcopenia or probable sarcopenia (2011-2020) were stratified by TyG-WHtR tertiles: T1 (≤4.30), T2 (4.30–5.01), and T3 (>5.01). To quantify the predictive utility of TyG-WHtR for CVD, methods such as Cox proportional hazards models, restricted cubic splines, and threshold regression analyses were utilized.

**Results:**

Over 7.3 years (median), incident CVD was documented in 727 individuals, including 258 patients who had a stroke and 560 patients who had heart diseases. Adjusted Cox models revealed that higher TyG-WHtR was independently associated with increased CVD risk: each one-unit increase corresponded to an 11% higher CVD risk (HR 1.11, 95% CI 1.01–1.22) and a 25% higher stroke risk (HR 1.25, 95% CI 1.06–1.47). Tertile analyses showed graded associations, with individuals in the highest tertile (T3) having a 51% higher risk of CVD, 90% higher risk of stroke, and 42% higher risk of heart diseases compared with T1. Threshold regression revealed a nonlinear relationship: when TyG-WHtR exceeded 3.76, CVD risk rose markedly (HR 1.22, *p* <.001), while below this threshold, lower TyG-WHtR levels were associated with reduced CVD risk (HR 0.80, *p* = .037).

**Discussion:**

In individuals with sarcopenia or probable sarcopenia, high TyG-WHtR is independently associated with higher CVD risk, demonstrating a distinct threshold effect. TyG-WHtR may be a valuable marker for predicting CVD risk in this population, thus enabling early cardiovascular risk stratification and personalized interventions for this group. However, these findings still require further validation through large-scale studies.

## Graphical Abstract

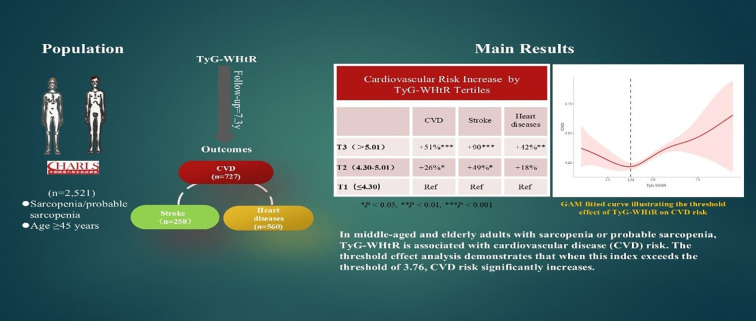

## 1 Introduction

Sarcopenia is an age-related geriatric syndrome characterized by the progressive loss of skeletal muscle mass, strength, and function. The prevalence of sarcopenia rises significantly as people age, and it has emerged as a prominent global public health issue due to the aging population (1, 2). Epidemiological data indicate that sarcopenia affects approximately 10%–18% of Chinese adults aged 60 years and older. An even larger proportion of older adults may be classified as having “probable sarcopenia,” a condition predicted a high risk of negative health events, notably falls, functional decline, frailty, and mortality (3, 4). As a critical geriatric condition, sarcopenia is associated with a considerably increased risk of all-cause mortality, and cardiovascular disease (CVD) is a leading cause of death in this population (5–7). Probable Sarcopenia, as a precursor stage of sarcopenia, is characterized by declined muscle strength and/or impaired physical performance despite not meeting the full diagnostic criteria for sarcopenia. This stage not only represents a high-risk status for sarcopenia progression but also serves as an independent risk factor for cardiovascular disease and all-cause mortality, distinct from obesity (8–10). Studies have demonstrated that both probable sarcopenia and confirmed sarcopenia are positively associated with CVD risk (9). Moreover, transitions in sarcopenia status influence CVD risk: progression from probable to confirmed sarcopenia further increases the risk, while reversion to non-sarcopenia status reduces it (10). The relationship between sarcopenia and CVD is multifactorial and involves several overlapping pathophysiological mechanisms, including chronic low-grade inflammation, insulin resistance (IR), oxidative stress, and endothelial dysfunction (6, 9, 11). Loss of muscle mass and strength impairs peripheral glucose utilization, leading to IR and subsequently atherosclerosis and hypertension. In turn, decreased muscle mass often results in lower physical activity and basal metabolic rate, increasing the likelihood of fat accumulation, dyslipidemia, and other metabolic disorders that accelerate CVD progression. Recently, increasing attention has been paid to the association between metabolic markers and CVD risk in individuals with sarcopenia to identify potential targets for improving care in middle-aged and older adults. Identifying simple, biologically meaningful predictors of cardiovascular risk in individuals with sarcopenia or probable sarcopenia is therefore of substantial clinical importance. Conducting such identification work specifically among individuals with probable sarcopenia holds particularly significant clinical value. This is because the pre-sarcopenia state is potentially reversible; timely intervention can effectively delay disease progression, improve patients’ functional status, and subsequently reduce the risk of cardiovascular events. This plays a crucial role in the health management of the elderly population. However, early screening tools for CVD risk in individuals with sarcopenia or probable sarcopenia remain limited, and relevant studies are scant.

Insulin resistance (IR), a pivotal link between metabolic disorders and cardiovascular injury, also actively participates in the development of sarcopenia (12, 13). Skeletal muscle is a primary site for insulin-mediated glucose uptake, accounting for approximately 80% of total glucose disposal. Thus, decreased muscle mass and impaired muscle function can disrupt glycogen synthesis and aggravate systemic IR. The triglyceride - glucose (TyG) index, a well-established proxy for IR, is associated with CVD risk. Triglyceride - glucose index combined with waist - to - height ratio (TyG - WHtR), a derivative index that combines TyG with the waist-to-height ratio, a composite measure integrating metabolic and anthropometric parameters, provides a more accurate reflection of visceral adiposity and CVD risk and is considered more practical for clinical risk stratification (14–16). But the connection between TyG-WHtR and CVD risk in elderly people with sarcopenia or probable sarcopenia hasn’t been well studied.

For the first time, this research investigates the association between TyG-WHtR and CVD incidence in individuals with sarcopenia or probable sarcopenia. These findings underscore the need for population-specific cardiovascular prevention strategies.

## 2 Methods

### 2.1 Study population

Data from five waves (2011–2020) of the nationally representative China Health and Retirement Longitudinal Study (CHARLS) database were used in this study. The CHARLS was constructed using a multistage probability sampling strategy to survey different populations from 150 local administrative units across 28 regions in China. The baseline response rate was 80.5%, ensuring representativeness of the Chinese individuals of middle and advanced age (17). This study was approved by the Institutional Review Board of Peking University (approval number: IRB00001052-11015). Written informed consent was obtained from all participants, and the study adhered to the Declaration of Helsinki and institutional ethical guidelines. CHARLS 2011 initially enrolled 17,708 participants, followed by survey waves conducted in 2013, 2015, 2018, and 2020.

**Figure 1:**
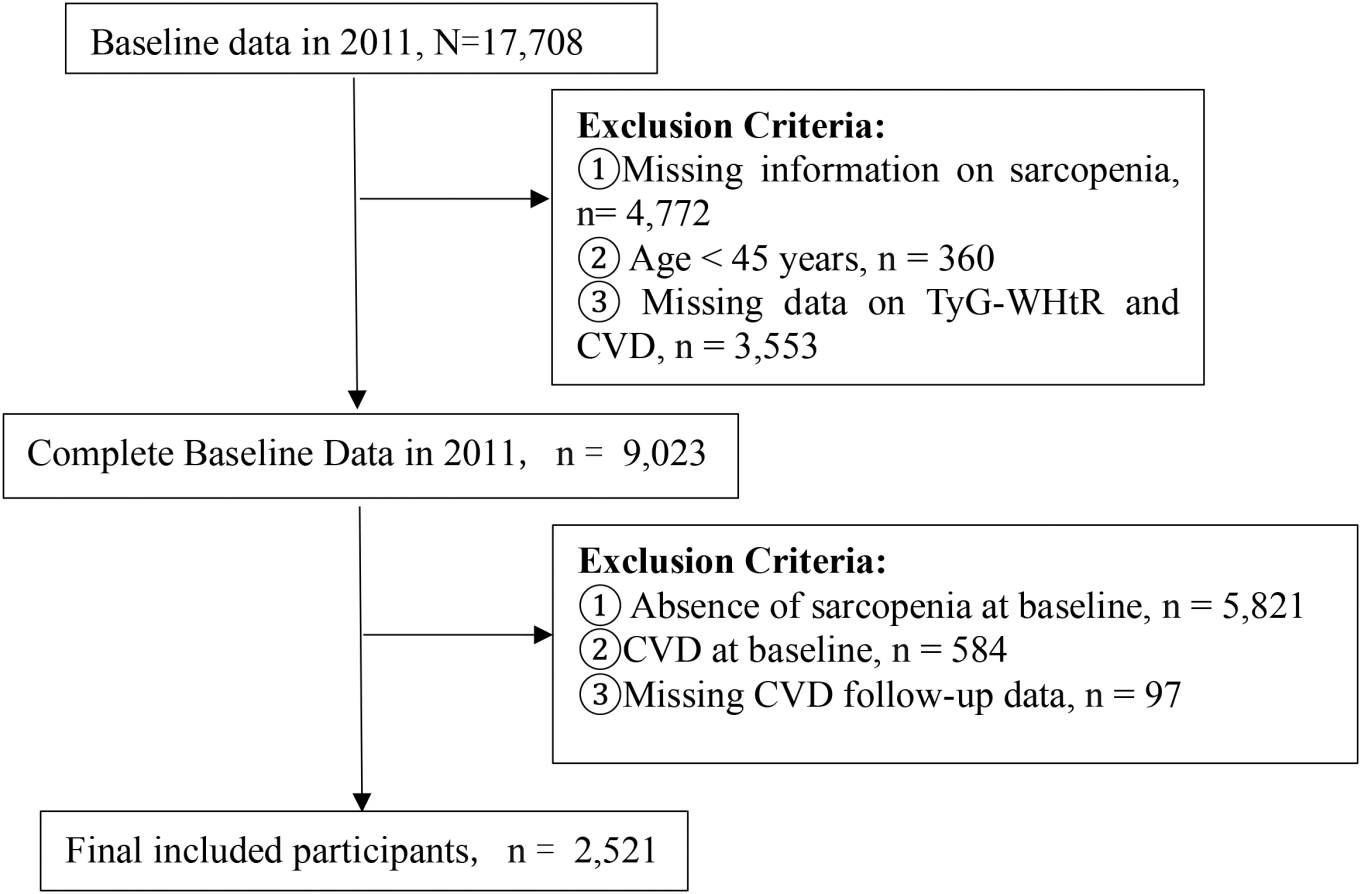
Flowchart of Participant Selection.

Participants were excluded if they (1) lacked sarcopenia status data in 2011, (2) were younger than 45 years, (3) had missing baseline TyG-WHtR or CVD data, (4) did not have sarcopenia at enrollment, (5) baseline CVD, or (6) CVD follow-up attrition (defined as missing at least one follow-up assessment). After the application of these criteria, a total of 2,521 participants were encompassed in the ultimate analysis (**Figure 1**).

### 2.2 Sarcopenia assessment criteria

Sarcopenia was evaluated based on the 2019 criteria established by the Asian Working Group for Sarcopenia (AWGS) (2). Recorded maximum grip strength from either hand. Low muscle strength thresholds were <28 kg in males and <18 kg in females. Appendicular skeletal muscle mass (ASM) was calculated using a validated prediction equation, and low muscle mass was defined as a height-adjusted ASM of <7.00 kg/m^2^ for men and <5.28 kg/m^2^ for women (18, 19). Low physical performance was defined as gait speed <1.0 m/s or chair stand ≥12 s. Probable sarcopenia: low strength or low performance; Sarcopenia: low mass plus low strength/performance (2).

#### 2.2.1 Assessment of ASM

ASM was calculated using the following formula: ASM = 0.193*W* + 0.107*H* − 4.157*G* − 0.037*A* − 2.631.

*where W = weight (kg), H = height (cm), G (1 for male and 2 for female), and A = age (years)*.

#### 2.2.2 Assessment of CVD events

CVD encompassed heart diseases and stroke. Consistent with previous studies (9, 20), incident CVD events were determined on the basis of participants’ self-reported histories of cardiovascular diagnoses.

#### 2.2.3 Calculation of TyG-WHtR

TyG-WHtR = Ln [(TG × FBG)/2] × (WC/height), with triglycerides (TG) and fasting blood glucose (FBG) expressed in mg/dL, and waist circumference (WC) and height in centimeters.

### 2.3 Definition of the follow-up duration

Follow-up spanned baseline to first CVD diagnosis or study endpoint.

### 2.4 Covariates

At the baseline survey, systematically trained investigators collected data by using structured questionnaires on demographic characteristics and health risk factors. The demographic characteristics analyzed were age, gender, education attainment, marital condition, residence type, height, weight, and WC. Health risk factors included body mass index (BMI), smoking status (yes/no), alcohol consumption (yes/no), hypertension, and diabetes. Hypertension was defined as systolic blood pressure (SBP) ≥140 mmHg, diastolic blood pressure (DBP) ≥90 mmHg, or current use of antihypertensive medications (21). Diabetes was defined as a self-reported physician diagnosis, use of antidiabetic medications, FBG ≥126 mg/dL, or glycated hemoglobin (HbA1c) ≥6.5% (22). The key biomarkers analyzed were FBG, total cholesterol (TC), TG, high-density lipoprotein cholesterol (HDL-C), low-density lipoprotein cholesterol (LDL-C), and C-reactive protein (CRP).

### 2.5 Statistical analysis

Analyses used R 4.1.3 and Stata 17.0. Normally-distributed continuous data were presented as mean ± SD; nonparametric variables as median (IQR). Continuous variables were compared using ANOVA for normally distributed data and the Kruskal–Wallis test for nonnormally distributed data, whereas categorical variables were compared using the χ² test.

Kaplan–Meier survival plots were made to display the cumulative incidence of CVD across TyG-WHtR tertiles. Associations between TyG-WHtR and CVD events were examined using Cox proportional hazards regression analysis. We formulated three models: Model 1 (unadjusted), Model 2 (adjusted for sex, age, educational attainment, marital condition, and residence type), and Model 3 (Model 2 plus smoking status, alcohol consumption, hypertension, diabetes, SBP, DBP, TC, and CRP).

Restricted cubic spline (RCS) analysis was employed to assess potential nonlinear relationships between the TyG-WHtR and CVD events. As a nonlinear relationship was detected, threshold effect analysis was performed by testing all possible cutoff points and selecting the most plausible value. A segmented Cox proportional hazards model was then used to estimate TyG-WHtR–outcome associations on either side of the threshold. The Generalized Additive Model (GAM) fitted curve visually demonstrated the association between TyG-WHtR and CVD. Subgroup analyses were performed to explore potential interactions by age (<60 vs. ≥60 years), sex, hypertension status, diabetes status, and sarcopenia status. Sensitivity analyses encompassing multiple imputation for missing data (23) and exclusion of extreme outliers to verify the robustness of the findings. Forest plots and violin plots were generated using GraphPad Prism version 9.0. P <.05 defined significance (two-sided).

## 3 Results

### 3.1 Participant demographics and clinical profiles

**Figure 2:**
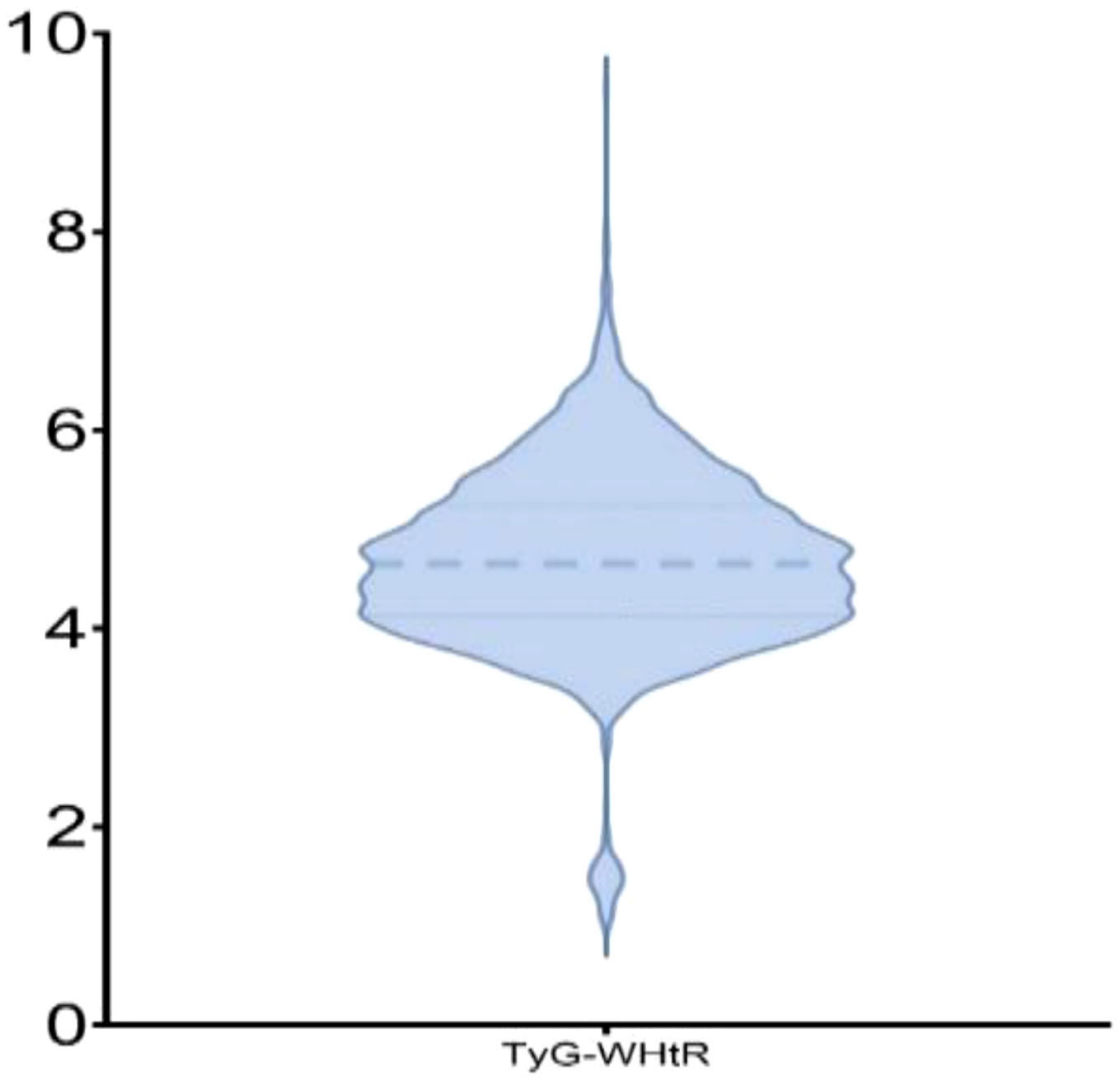
Violin plot depicting the distribution of TyG-WHtR. The thick dashed line represents the median of TyG-WHtR, while the thin dashed lines above and below indicate the 25th and 75th percentiles of TyG-WHtR, respectively.

The cohort included 2,521 participants (mean age, 63 ± 9.95 years; 59.06% women). On the basis of TyG-WHtR tertiles, participants were categorized into T1–T3 groups. In contrast to the T1 group, the T3 group had significantly higher proportions of women (77.53% vs. 43.45%), urban residents (36.03% vs. 25.00%), individuals with hypertension (60.43% vs. 31.21%), and those with diabetes (29.25% vs. 8.33%; all *p* <.001). With increasing TyG-WHtR levels, blood pressure, FBG, TC, TG, LDL-C, and CRP levels significantly increased, whereas HDL-C levels significantly decreased (all *p* <.001; **Table 1**). The distribution of TyG-WHtR is illustrated in the violin plot (**Figure 2**).

**Table 1:** Baseline Characteristics: TyG-WHtR T1-T3.

TyG-WHtR tertile	T1(≤4.30)	T2(4.30-5.01)	T3(>5.01)	*p*-value
n	840	840	841	
Age(years)	62.59 ± 9.97	62.99 ± 10.09	63.41 ± 9.77	0.236
Sex(n, %)				**<0.001**
Male	475 (56.55%)	368 (43.81%)	189 (22.47%)	
Female	365 (43.45%)	472 (56.19%)	652 (77.53%)	
Marriage(n, %)				0.135
Married	709 (84.40%)	689 (82.02%)	679 (80.74%)	
Others	131 (15.60%)	151 (17.98%)	162 (19.26%)	
Education level(n, %)				0.521
Below primary school	505 (60.12%)	507 (60.36%)	538 (63.97%)	
Primary school	179 (21.31%)	173 (20.60%)	158 (18.79%)	
Secondary school	105 (12.50%)	118 (14.05%)	104 (12.37%)	
High school or above	51 (6.07%)	42 (5.00%)	41 (4.88%)	
Residence Type(n, %)				**< 0.001**
Rural	630 (75.00%)	611 (72.74%)	538 (63.97%)	
Urban	210 (25.00%)	229 (27.26%)	303 (36.03%)	
Drinking(n, %)				**< 0.001**
No	517 (61.55%)	605 (72.02%)	689 (81.93%)	
Yes	323 (38.45%)	235 (27.98%)	152 (18.07%)	
Smoking*(n, %)				**< 0.001**
No	496 (59.47%)	617 (73.54%)	715 (85.63%)	
Yes	338 (40.53%)	222 (26.46%)	120 (14.37%)	
BMI (kg/m^2^)	20.35 ± 2.76	22.78 ± 2.83	26.26 ± 3.73	**< 0.001**
HBP* (n, %)				**< 0.001**
No	573 (68.79%)	499 (59.62%)	332 (39.57%)	
Yes	260 (31.21%)	338 (40.38%)	507 (60.43%)	
Diabetes(n, %)				**< 0.001**
No	770 (91.67%)	718 (85.48%)	595 (70.75%)	
Yes	70 (8.33%)	122 (14.52%)	246 (29.25%)	
SBP* (mmHg)	125.98 ± 22.57	130.12 ± 21.14	138.15 ± 22.62	**< 0.001**
DBP* (mmHg)	72.44 ± 12.53	74.21 ± 11.60	78.18 ± 12.47	**< 0.001**
FBG (mg/dL)	100.68 ± 18.91	109.07 ± 37.41	123.91 ± 52.62	**< 0.001**
TC (mg/dL)	182.06 ± 35.49	192.44 ± 34.20	203.71 ± 39.96	**< 0.001**
TG (mg/dL)	75.22(58.41-97.35)	103.55 (77.88-136.51)	151.34 (110.62-210.63)	**< 0.001**
HDL-C (mg/dL)	57.56 ± 14.91	51.81 ± 13.99	44.63 ± 12.89	**< 0.001**
LDL-C (mg/dL)	109.46 ± 31.52	119.00 ± 30.84	121.15 ± 39.02	**< 0.001**
CRP (mg/dL)	0.82 (0.49-1.94)	0.95 (0.54-1.98)	1.46 (0.82-2.88)	**< 0.001**
TyG-WHtR	3.78 ± 0.62	4.65 ± 0.21	5.64 ± 0.51	**< 0.001**

*Missing values: Smoking (n=13, 0.52%), hypertension (n=12, 0.48%), SBP (n=24), DBP (n=25).

BMI, body mass index; HBP, high blood pressure; SBP, systolic blood pressure; DBP, diastolic blood pressure; FBG, fasting blood glucose; TC, total cholesterol; TG, triglyceride; HDL‐C, high‐density lipoprotein cholesterol; LDL-C, low-density lipoprotein cholesterol; CRP, C-reactive protein.

Bold values denote statistical significance (*P* < 0.05).

### 3.2 Relationship between the TyG-WHtR index and CVD events in patients with sarcopenia or probable sarcopenia

**Figure 3:**
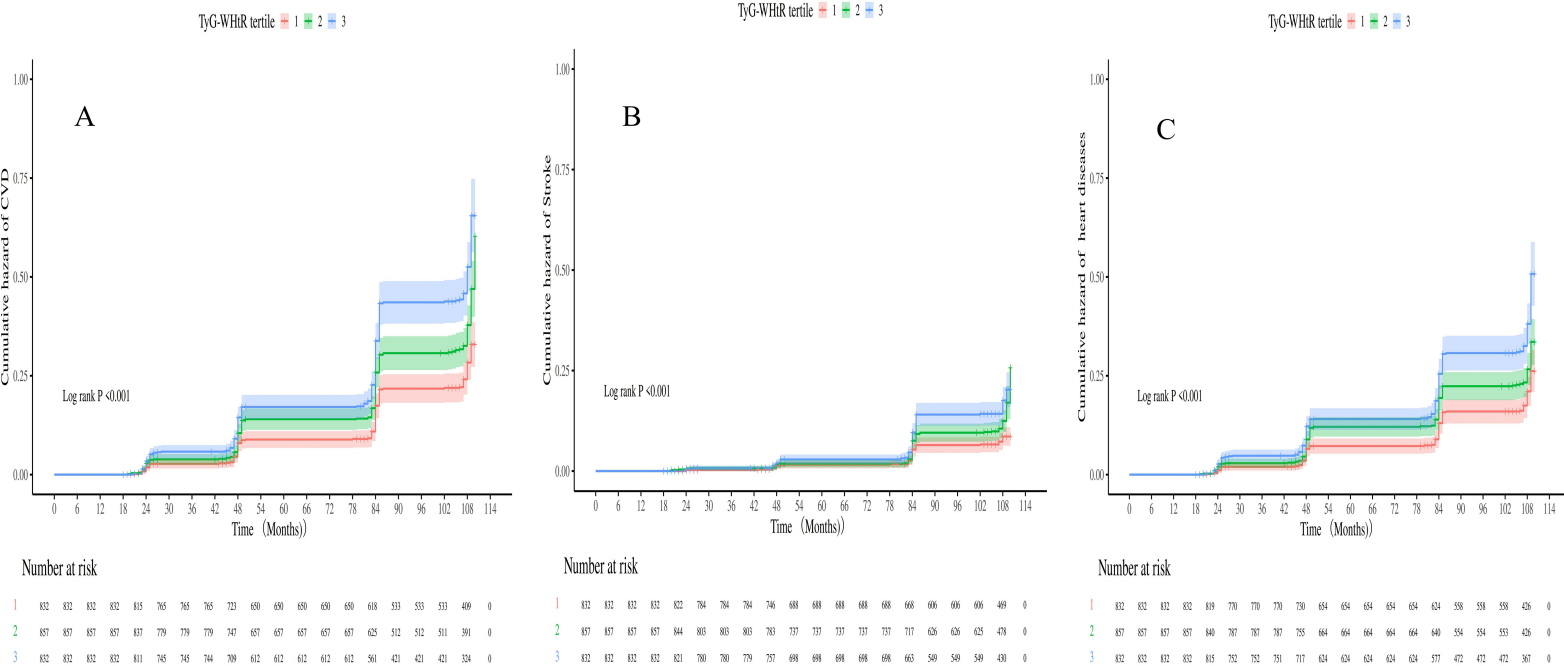
**(A-C)** Kaplan-Meier curves for CVD, heart diseases and stroke stratified by TyG-WHtR index tertiles.

Over 7.3 years (median), 727 incident CVD events were documented, comprising 560 heart diseases and 258 strokes. The annual incidence rates were 395 per 10,000 person-years (3.95%; 95% CI: 3.75%–4.16%) for CVD, 140 per 10,000 person-years (1.40%; 95% CI: 1.28%–1.53%) for stroke, and 304 per 10,000 person-years (3.04%; 95% CI: 2.87%–3.23%) for heart diseases. Kaplan–Meier survival analysis (**Figure 3**) demonstrated a significantly higher CVD incidence in individuals with sarcopenia or probable sarcopenia and high TyG-WHtR levels (log-rank *p* <.001).

Cox models assessed TyG-WHtR–CVD association in sarcopenia or probable sarcopenia (**Table 2**). In the completely adjusted model, when TyG - WHtR went up by one unit, there was an 11% greater risk of CVD (HR = 1.11, 95% CI: 1.01–1.22; *p* = .024). A graded association was observed across TyG-WHtR tertiles, with participants in T3 exhibiting a 51% higher CVD risk than did those in T1 (HR = 1.51, 95% CI: 1.23–1.87). For stroke, a similar pattern was observed: **e**ach TyG-WHtR unit increase elevated risk (HR = 1.25, 95% CI: 1.06–1.47; *p* = .008), and the T3 group exhibited 90% excess stroke risk vs. T1 (HR = 1.90, 95% CI: 1.32–2.74; *p* = .002). Furthermore, the unadjusted model indicated a significant association between TyG-WHtR and heart diseases risk (HR = 1.25, 95% CI: 1.14–1.37), but the effect size tended to decrease after multivariable adjustment (fully adjusted model HR = 1.05; *p* = .128). However, when analyzed by tertiles, T3 remained significantly associated with a higher heart diseases risk (HR = 1.42, 95% CI: 1.12–1.80; *p* = .004).

**Table 2:** Association between baseline TyG-WHtR index and CAS events in sarcopenia or possible sarcopenia patients.

Outcome		Model1 HR (95% CI)	*P*-value	Model2 HR (95% CI)	*P*-value	Model3 HR (95% CI)	*P*-value
CVD	Continuous TyG-WHtR	1.26 (1.16, 1.37)	<0.001	1.20 (1.10, 1.31)	<0.001	1.11 (1.01, 1.22)	**0.024**
	Q1	1.00		1.00		1.00	
	Q2	1.38 (1.13, 1.67)	0.001	1.34 (1.10, 1.62)	0.004	1.26 (1.03, 1.54)	**0.023**
	Q3	1.88 (1.56, 2.26)	<0.001	1.75 (1.44, 2.12)	<0.001	1.51 (1.23, 1.87)	**<0.001**
Stroke	Continuous TyG-WHtR	1.33 (1.16, 1.53)	<0.001	1.34 (1.16, 1.55)	<0.001	1.25 (1.06, 1.47)	**0.008**
	Q1	1.00		1.00		1.00	
	Q2	1.60 (1.14, 2.23)	<0.001	1.62 (1.16, 2.28)	0.005	1.49 (1.06, 2.11)	**0.023**
	Q3	2.11 (1.53, 2.91)	<0.001	2.23 (1.59, 3.13)	<0.001	1.90(1.32, 2.74)	**0.002**
Heart diseseases	Continuous TyG-WHtR	1.25 (1.14, 1.37)	<0.001	1.16 (1.06, 1.28)	0.002	1.08 (0.98, 1.20)	0.128
	Q1	1.00		1.00		1.00	
	Q2	1.31 (1.05, 1.63)	0.017	1.24 (1.00, 1.56)	0.054	1.18 (0.94, 1.49)	0.146
	Q3	1.83 (1.49, 2.26)	<0.001	1.61 (1.29, 2.00)	<0.001	1.42 (1.12, 1.80)	**0.004**

Model 1: Crude; Model 2: Adjusted (sex, age, education, marriage, residence type); Model 3: Model 2 + smoking, alcohol consumption, hypertension, diabetes, SBP, DBP, TC, CRP.

Bold values denote statistical significance (*P* < 0.05).

**Figure 4:**
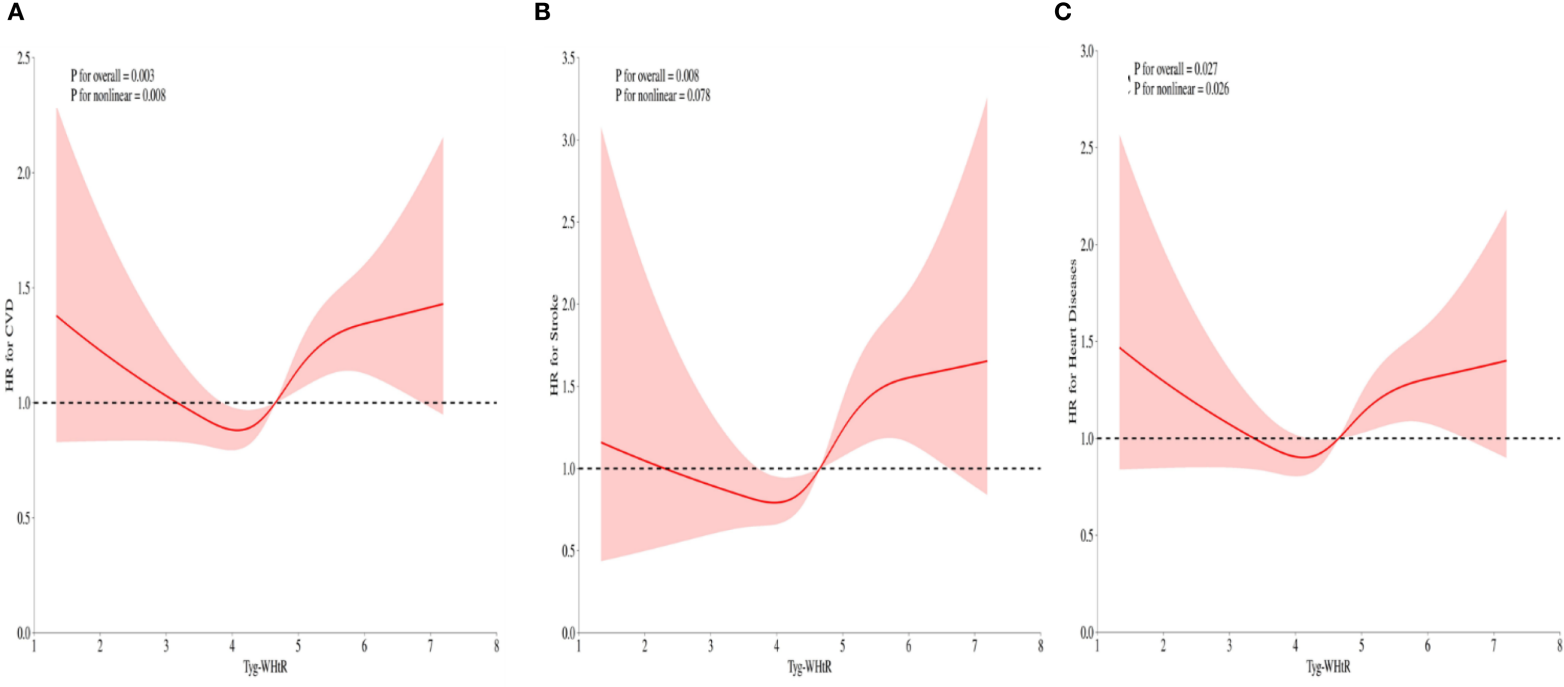
**(A-C)** RCS illustrating the relationship between the TyG-WHtR index and CVD, stroke, and heart diseases in individuals with sarcopenia or probable sarcopenia. The y-axis represents the HR (95% CI), whereas the x-axis displays TyG-WHtR index values. The model was adjusted for sex, age, education level, marital status, residence type, smoking status, alcohol consumption, hypertension, diabetes, SBP, DBP, TC, and CRP. The solid line and shaded area denote the estimated values and their corresponding 95% CIs, respectively.

After full adjustment for covariates, RCS curves demonstrated a nonlinear regression between TyG-WHtR and CVD events in individuals with sarcopenia or probable sarcopenia (**Figures 4A–C**). Significant associations were observed between TyG-WHtR and the probability of CVD occurrence (all *p*-overall <.05). U-shaped relationships were observed for CVD, stroke, and heart diseases. The nonlinear association was statistically significant for CVD (*p*-nonlinear = .008) and heart diseases (*p*-nonlinear = .026). For stroke, the nonlinear trend was not statistically significant (*p* = .078), although the U-shaped pattern was similar. Additional analysis showed that the link between TyG - WHtR and CVD was non - significant in those with low muscle mass/strength but significant in those with low physical function (all *p*-overall <.05; **Supplementary Figures S1**-**S3**).

### 3.3 Threshold analysis of TyG-WHtR in relation to cardiovascular risk among patients with sarcopenia

**Figure 5:**
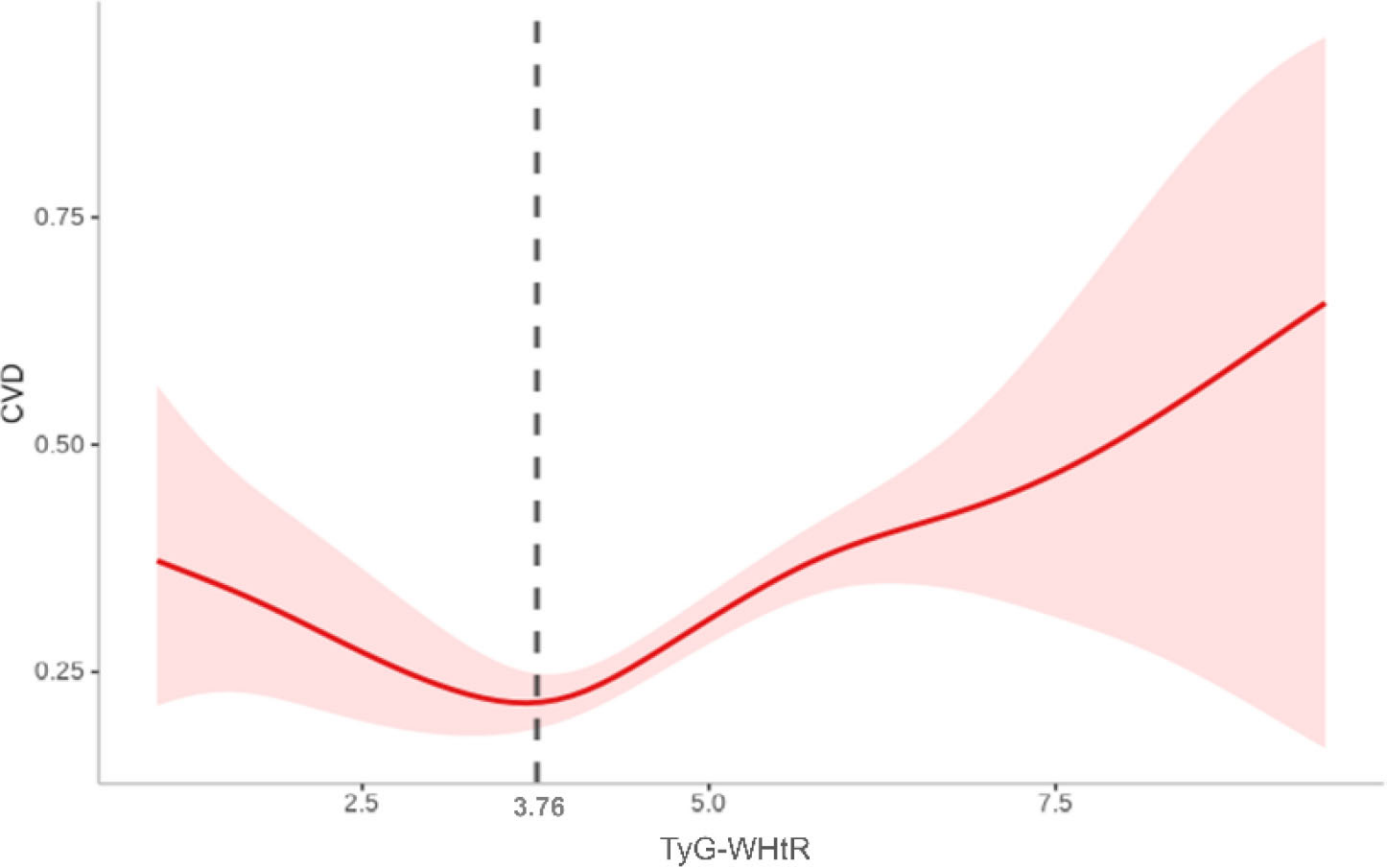
Nonlinear association between TyG-WHtR and CVD based on generalized additive model (GAM). A distinct threshold effect was observed at a TyG-WHtR value of 3.76 (as indicated by the vertical dashed black line).

Nonlinear TyG-WHtR–CVD associations detected by using threshold regression models (**Table 3**). Significant threshold effects were observed for overall CVD and heart diseases, as confirmed by likelihood ratio tests (*p* = .003 and *p* = .010, respectively). When the TyG-WHtR index exceeded the critical threshold (3.76 for CVD and 3.74 for heart diseases), the risk increased significantly. Above these thresholds, overall CVD risk increased by 22% (HR = 1.22, 95% CI: 1.10–1.36, *p* <.001) and heart diseases risk increased by 19% (HR = 1.19, 95% CI: 1.05–1.34, *p* = .005). By contrast, below the CVD threshold, TyG-WHtR exhibited an inverse correlation with overall CVD risk (HR = 0.80, 95% CI: 0.65–0.99, *p* = .037). The GAM curve fitting plot revealed a distinct nonlinear relationship between TyG-WHtR and CVD risk, with a clear threshold effect observed at TyG-WHtR = 3.76, beyond which CVD risk increases significantly (**Figure 5**).

**Table 3:** TyG-WHtR CVD Risk Thresholds in individuals with sarcopenia or possible sarcopenia.

Outcome	Adjusted HR (95% CI), *p*-value
Overall CVD	
Cutoff	3.76
TyG-WHtR<3.76	0.80 (0.65, 0.99), **0.037**
TyG-WHtR>3.76	1.22 (1.10, 1.36), **< 0.001**
Likelihood Ratio Test	0.003
Heart Diseases	
Cutoff	3.74
TyG-WHtR<3.74	0.79 (0.62, 1.00), **0.048**
TyG-WHtR>3.74	1.19 (1.05, 1.34), **0.005**
Likelihood Ratio Test	0.010

Hazard ratios were estimated using Cox proportional hazards regression models, with full adjustment for the following covariates: sex, age, education level, marital status, residence type, smoking status, alcohol consumption status, hypertension, diabetes, SBP, DBP, TC, and CRP.

Bold values denote statistical significance (*P* < 0.05).

### 3.4 Subgroup analysis

**Figure 6:**
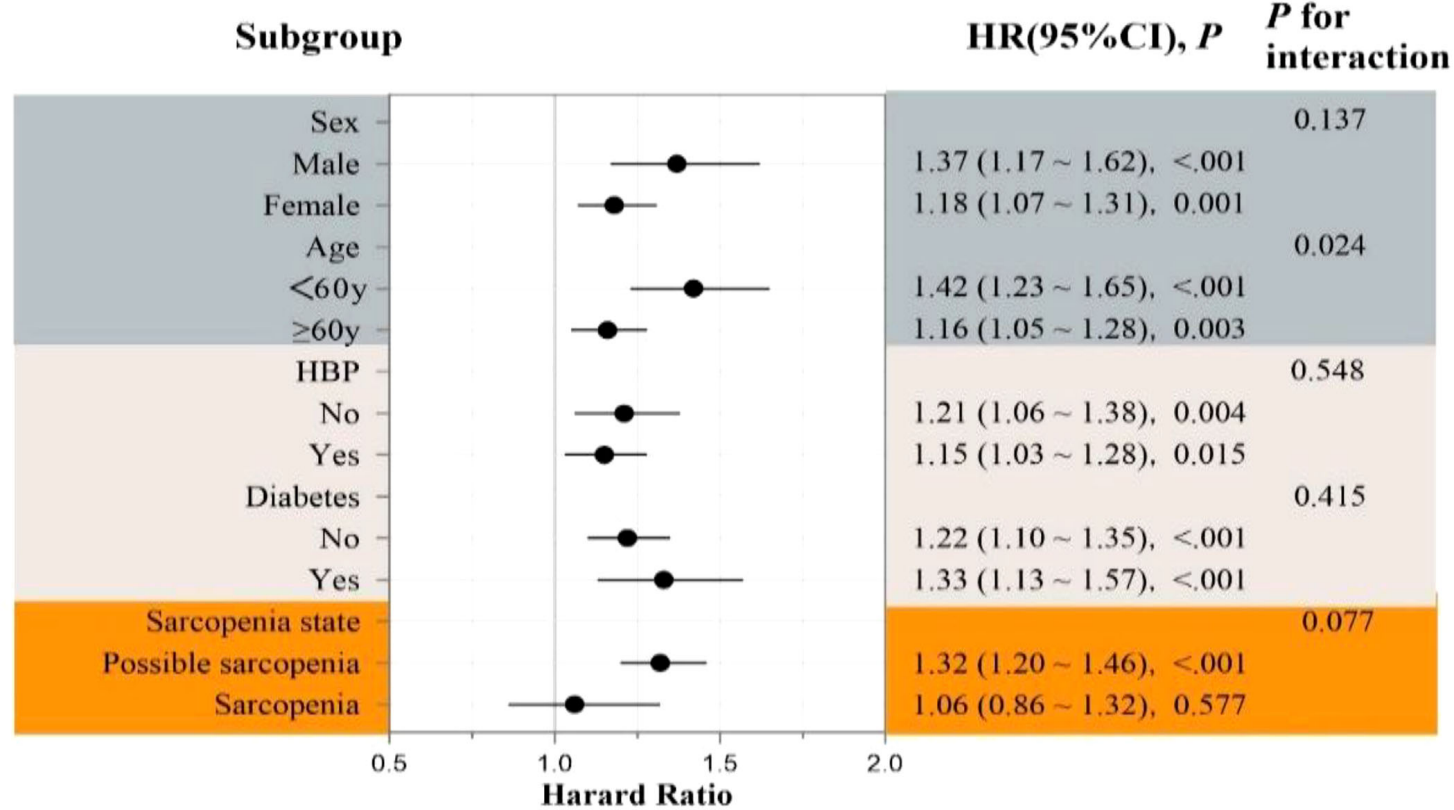
Forest plot of subgroup analyses for the association between TyG-WHtR index and incident CVD risk.

TyG-WHtR-CVD association persisted across subgroups defined by sex, hypertension, diabetes, and sarcopenia status (*p*-interaction = .137,.548,.415, and.077, respectively). Notably, participants younger than 60 years revealed a more robust association (HR = 1.42, 95% CI: 1.23–1.65), with a significant interaction by age (*p* = .024), indicating a greater risk in younger individuals (**Figure 6**).

### 3.5 Sensitivity analysis

After missing data were filled in through multiple imputation, the association between higher TyG-WHtR levels and increased CVD risk remained significant among participants with sarcopenia or probable sarcopenia (**Supplementary Table S1**). This correlation persisted after the exclusion of extreme TyG-WHtR outliers (n = 45; **Supplementary Table S2**).

## 4 Discussion

This study is the first to identify a significant dose–response relationship between TyG-WHtR and CVD risk in individuals with sarcopenia or probable sarcopenia. As TyG-WHtR increases, the risk of overall CVD exhibits an upward trend. Threshold analysis indicates that when TyG-WHtR exceeds the critical threshold (3.76), CVD risk increases significantly, whereas below this threshold, the risk remains stable or decreases.

Multiple studies (14, 24, 25) have reported that TyG-WHtR outperforms the TyG index in predicting CVD risk, demonstrating superior predictive accuracy and stronger associations with cardiovascular mortality. Dang et al. (14) determined that TyG-WHtR exhibited the greatest HR (1.66, 95% CI: 1.21–2.29) for predicting CVD mortality, establishing it as the strongest predictor. By contrast, the TyG index had weaker or nonsignificant associations with CVD incidence and mortality. In the general population, TyG-WHtR and TyG-WC were significantly and positively correlated with CVD mortality, whereas the TyG index unassociated with CVD mortality (14, 25). Our findings in patients with sarcopenia or probable sarcopenia are consistent with this upward trend, with TyG-WHtR associated with higher CVD risk (HR = 1.11, 95% CI: 1.01–1.22). Moreover, TyG-WHtR outperformed the TyG index in integrated discrimination improvement (IDI), C-index, and net reclassification index (NRI) analyses, confirming its superior ability to stratify CVD risk and predict mortality (24, 25). This enhanced predictive value of TyG-WHtR for CVD is largely attributable to the incorporation of WHtR, which more accurately reflects visceral fat distribution and metabolic dysfunction.

TyG-WHtR demonstrates population-dependent variability in predicting CVD risk, with notable differences observed in individuals with fatty liver disease or diabetes. In populations with fatty liver–related conditions, TyG-WHtR retains strong predictive value. For instance, in these patients, TyG-WHtR significantly predicts cardiovascular mortality, particularly in younger individuals and those without complications (26). Our subgroup analysis further supports this evidence: among younger participants with sarcopenia, higher TyG-WHtR was associated with a 42% increased risk of CVD (HR = 1.42, 95% CI: 1.23–1.65) after full adjustment. In nondiabetic populations, TyG-WHtR was also significantly and positively associated with heart diseases risk (HR = 1.34, 95% CI: 1.13–1.60) (27). However, in patients with type 2 diabetes mellitus (T2DM), TyG-WHtR, TyG, and TyG-WC were not significantly associated with heart diseases risk; only TyG-BMI reached significance in the highest quartile (HR = 1.86, 95% CI: 1.02–3.40) (27). Similarly, in our sarcopenic cohort, TyG-WHtR was not significantly associated with heart diseases risk. By contrast, multiple cohort studies have reported that TyG-WHtR is significantly associated with stroke risk regardless of diabetes status (26, 28, 29), Consistent with these findings, we observed a significant relationship between TyG-WHtR and stroke risk (HR = 1.25, 95% CI: 1.06–1.47), with directionally consistent associations in both diabetic and nondiabetic subgroups.

In individuals with sarcopenia or probable sarcopenia, the relationship between TyG-WHtR and CVD risk is likely driven by metabolic dysregulation and persistent low-grade inflammation. The TyG index is a well-established marker for IR, whereas WHtR reflects visceral fat accumulation. Combining these parameters enables a more comprehensive evaluation of disturbances in glucose and lipid metabolism. IR impairs glucose uptake, suppresses muscle protein synthesis, and promotes proteolysis, thereby accelerating muscle mass loss and potentially initiating or worsening sarcopenia. Concurrently, excessive visceral fat contributes to the upregulation of proinflammatory cytokines, creating a chronic low-grade inflammatory state that plays a critical role in both CVD pathogenesis and sarcopenia progression (30–33).

In patients with sarcopenia, these metabolic and inflammatory disturbances may be further exacerbated. Mitochondrial malfunction, frequently encountered in the skeletal muscle of individuals with sarcopenia, impairs energy metabolism and reduces insulin sensitivity, thereby creating a vicious cycle with increased TyG levels that further disrupt metabolic homeostasis (34). In addition, sarcopenia is often accompanied by decreased physical activity and basal metabolic rate, which promote fat accumulation and worsen IR, ultimately leading to “sarcopenic obesity” (35, 36). Such individuals may exhibit marked visceral adiposity and metabolic abnormalities even when BMI remains within the normal range. In these cases, TyG-WHtR may more effectively identify high-risk individuals with subclinical metabolic dysfunction than BMI, indicating its superior clinical utility (6).

The current study revealed a nonlinear association between TyG-WHtR and cardiovascular disease risk, characterized by a distinct threshold effect. When TyG-WHtR exceeds 3.76, CVD risk increases substantially. This threshold may represent a tipping point in the progression of IR and lipid metabolism disorders, beyond which more atherogenic lipoprotein profiles, endothelial dysfunction, and systemic inflammation are triggered, accelerating atherosclerosis (37–39). Increased TyG-WHtR may also promote oxidative stress, further aggravating myocardial injury. By contrast, when TyG-WHtR remains below the threshold, cardiovascular risk appears to stabilize at a lower level, likely due to preserved insulin sensitivity, decreased visceral fat burden, and more favorable metabolic and cardiovascular profiles (40–42).

The core innovation of this study lies in being the first to identify a dose-response relationship between TyG-WHtR and cardiovascular disease risk in individuals with sarcopenia or probable sarcopenia. Sarcopenia involves not only loss of muscle function but also low-grade inflammation and metabolic dysregulation, creating a “dual-risk” background for CVD. Our study innovatively introduces TyG-WHtR, a composite index combining insulin resistance (TyG) and visceral obesity (WHtR), which better captures the complex pathophysiology driving CVD risk in this population. A distinct risk threshold (3.76) was identified, transforming generalized risk perception into an actionable clinical tool. Exceeding this threshold signals the need for intensified management, including glucose/lipid monitoring and personalized lifestyle interventions. TyG-WHtR was also significantly associated with stroke risk and showed higher predictive value in younger subgroups. As a simple, low-cost, and non-invasive marker, TyG-WHtR is highly suitable for primary care and geriatrics. It promotes integrated management of muscular and cardiometabolic health, providing a practical tool for improving long-term outcomes in the growing sarcopenia population.

This study has several limitations. First, skeletal muscle mass was not measured using gold-standard techniques, such as dual-energy X-ray absorptiometry, which may have introduced systematic bias. Second, although we adjusted for common CVD risk factors, including age, sex, smoking history, and hypertension, residual confounding from unmeasured variables, such as dietary patterns, cannot be excluded. Third, although we identified a TyG-WHtR threshold for CVD risk in individuals with sarcopenia, its generalizability and clinical applicability should be confirmed in larger, prospective cohort studies involving more diverse populations. Moreover, the cohort in our study was composed solely of adults (≥45 years), which may limit the applicability of the findings beyond studied population characteristics. Finally, caution is warranted when comparing our results with those of studies that apply different sarcopenia diagnostic criteria, such as the European Working Group on Sarcopenia in Older People guidelines, instead of the AWGS criteria used here.

## 5 Conclusions

Our results imply that TyG-WHtR is a good predictor of CVD risk for those individuals with sarcopenia or probable sarcopenia. Measuring TyG-WHtR may therefore assist in risk stratification and prognosis assessment in this high-risk population. Future studies should explore whether targeted interventions designed to reduce TyG-WHtR can lead to improved clinical outcomes in these patients.

## Data Availability

The raw data supporting the conclusions of this article will be made available by the authors, without undue reservation.

## Supplementary material

Supplementary Figure 1 A–C RCS curves depicting the association between the TyG-WHtR index and the risks of CVD, stroke, and heart diseases among participants with low muscle mass. The y-axis represents the HR (95% CI), whereas the x-axis displays TyG-WHtR index values. The model was adjusted for sex, age, education level, marital status, residence type, smoking status, alcohol consumption, hypertension, diabetes, SBP, DBP, TC, and CRP. The solid line and shaded area denote the estimated values and their corresponding 95% CIs, respectively.

Supplementary Figure 2 A–C RCS curves illustrating the association between the TyG-WHtR index and the risks of CVD, stroke, and heart diseases among participants with low muscle strength. The y-axis represents the HR (95% CI), whereas the x-axis displays TyG-WHtR index values. The model was adjusted for sex, age, education level, marital status, residence type, smoking status, alcohol consumption, hypertension, diabetes, SBP, DBP, TC, and CRP. The solid line and shaded area denote the estimated values and their corresponding 95% CIs, respectively.

Supplementary Figure 3 A–C RCS curves illustrating the association between the TyG-WHtR index and the risks of CVD, stroke, and heart diseases among participants with low physical performance. The y-axis represents HR (95% CI), whereas the x-axis displays TyG-WHtR index values. The model was adjusted for sex, age, education level, marital status, residence type, smoking status, alcohol consumption, hypertension, diabetes, SBP, DBP, TC, and CRP. The solid line and shaded area denote the estimated values and their corresponding 95% CIs, respectively.

Supplementary Table 1 Correlation between TyG-WHtR and overall CVD, stroke, and heart diseases in individuals with sarcopenia or possible sarcopenia after multiple imputation for missing data. Model 1: Crude; Model 2: Adjusted (sex, age, education, marriage, residence type); Model 3: Model 2 + smoking, alcohol consumption, hypertension, diabetes, SBP, DBP, TC, CRP.

Supplementary Table 2 Correlation between TyG-WHtR and overall CVD, stroke, and heart diseases in individuals with sarcopenia or possible sarcopenia after excluding extreme outliers. Model 1: Crude; Model 2: Adjusted (sex, age, education, marriage, residence type); Model 3: Model 2 + smoking, alcohol consumption, hypertension, diabetes, SBP, DBP, TC, CRP.
