# Supplementary material for: Association between the triglyceride-glucose-waist-to-height ratio and cardiovascular disease in Chinese adults with sarcopenia or probable sarcopenia
Source: Front Endocrinol (Lausanne). 2025 Oct 13;16:1686885. doi: 10.3389/fendo.2025.1686885 (PMC12554443; doi:10.3389/fendo.2025.1686885)
Supplement: Supplementary Figure 1 — A–C RCS curves depicting the association between the TyG-WHtR index and the risks of CVD, stroke, and heart diseases among participants with low muscle mass. The y-axis represents the HR (95% CI), whereas the x-axis displays TyG-WHtR index values. The model was adjusted for sex, age, education level, marital status, residence type, smoking status, alcohol consumption, hypertension, diabetes, SBP, DBP, TC, and CRP. The solid line and shaded area denote the estimated values and their corresponding 95% CIs, respectively. [file DataSheet1.docx]

**Table S1** Correlation between TyG-WHtR and overall CVD, stroke, and heart diseases in individuals with sarcopenia or possible sarcopenia after multiple imputation for missing data

|  |  | **Model1**  **HR (95% CI)** | ***P*-value** | **Model2**  **HR (95% CI)** | ***P*-value** | **Model3**  **HR (95% CI)** | ***p*-value** |
| --- | --- | --- | --- | --- | --- | --- | --- |
| CVD | Continuous TyG-WHtR | 1.26 (1.16, 1.37) | <0.001 | 1.20 (1.10, 1.31) | <0.001 | 1.11 (1.01, 1.22) | **0.030** |
|  | T1 | 1.00 |  | 1.00 |  | 1.00 |  |
|  | T2 | 1.38 (1.14, 1.68) | 0.001 | 1.34 (1.10, 1.63) | 0.003 | 1.25 (1.03, 1.52) | **0.026** |
|  | T3 | 1.88 (1.56, 2.26) | <0.001 | 1.75(1.44, 2.11) | <0.001 | 1.50 (1.22, 1.85) | **0.001** |
| Stroke | Continuous TyG-WHtR | 1.33 (1.16, 1.53) | <0.001 | 1.34 (1.16, 1.55) | <0.001 | 1.23(1.05, 1.44) | **0.010** |
|  | T1 | 1.00 |  | 1.00 |  | 1.00 |  |
|  | T2 | 1.60 (1.14, 2.23) | 0.006 | 1.62 (1.15, 2.27) | 0.005 | 1.51 (1.07, 2.13) | **0.019** |
|  | T3 | 2.11 (1.53, 2.91) | <0.001 | 2.23 (1.59, 3.13) | <0.001 | 1.88 (1.31, 2.70) | **<0.001** |
| Heart diseases | Continuous TyG-WHtR | 1.25 (1.14, 1.37) | <0.001 | 1.16 (1.06, 1.28) | 0.002 | 1.09 (0.98, 1.21) | 0.109 |
|  | T1 | 1.00 |  | 1.00 |  | 1.00 |  |
|  | T2 | 1.31 (1.05, 1.63) | 0.017 | 1.24 (1.00, 1.56) | 0.054 | 1.17 (0.94, 1.47) | 0.164 |
|  | T3 | 1.83 (1.49, 2.26) | <0.001 | 1.61 (1.29, 2.00) | <0.001 | 1.41 (1.12, 1.78) | **0.004** |

Model 1: Crude;
Model 2: Adjusted (sex, age, education, marriage, residence type);
Model 3**:** Model 2 + smoking, alcohol consumption, hypertension, diabetes, SBP, DBP, TC, CRP.

Bold values denote statistical significance (*P* < 0.05).

**Table S2** Correlation between TyG-WHtR and overall CVD, stroke, and heart diseases in individuals with sarcopenia or possible sarcopenia after excluding extreme outliers

|  |  | **Model1**  **HR (95% CI)** | ***P*-value** | **Model2**  **HR (95% CI)** | ***P*-value** | **Model3**  **HR (95% CI)** | ***p*-value** |
| --- | --- | --- | --- | --- | --- | --- | --- |
| CVD | Continuous TyG-WHtR | 1.35 (1.23, 1.47) | <0.001 | 1.29 (1.17, 1.42) | <0.001 | 1.20 (1.08, 1.34) | **< 0.001** |
|  | T1 | 1.00 |  | 1.00 |  | 1.00 |  |
|  | T2 | 1.43 (1.17, 1.74) | 0.004 | 1.39 (1.14, 1.69) | 0.001 | 1.31 (1.07, 1.61) | **0.009** |
|  | T3 | 1.94(1.60, 2.34) | <0.001 | 1.81 (1.48, 2.21) | <0.001 | 1.59 (1.28, 1.77) | **< 0.001** |
| Stroke | Continuous TyG-WHtR | 1.38 (1.19, 1.60) | <0.001 | 1.41 (1.20, 1.64) | <0.001 | 1.33 (1.12, 1.59) | **0.014** |
|  | T1 | 1.00 |  | 1.00 |  | 1.00 |  |
|  | T2 | 1.59 (1.14, 2.22) | 0.007 | 1.62 (1.16, 2.27) | 0.005 | 1.53 (1.08, 2.17) | **0.017** |
|  | T3 | 2.03 (1.47, 2.80) | <0.001 | 2.15 (1.53, 3.03) | <0.001 | 1.90(1.31, 2.76) | **< 0.001** |
| Heart diseases | Continuous TyG-WHtR | 1.25 (1.14, 1.37) | <0.001 | 1.16 (1.06, 1.28) | 0.002 | 1.08 (0.98, 1.20) | 0.128 |
|  | T1 | 1.00 |  | 1.00 |  | 1.00 |  |
|  | T2 | 1.41 (1.12, 1.77) | 0.003 | 1.34 (1.07, 1.69) | 0.011 | 1.27 (1.00, 1.60) | **0.047** |
|  | T3 | 1.96 (1.58, 2.43) | <0.001 | 1.73 (1.38, 2.17) | <0.001 | 1.53 (1.20, 1.96) | **< 0.001** |

Model 1: Crude;
Model 2: Adjusted (sex, age, education, marriage, residence type);
Model 3**:** Model 2 + smoking, alcohol consumption, hypertension, diabetes, SBP, DBP, TC, CRP.

Bold values denote statistical significance (*P* < 0.05).

**
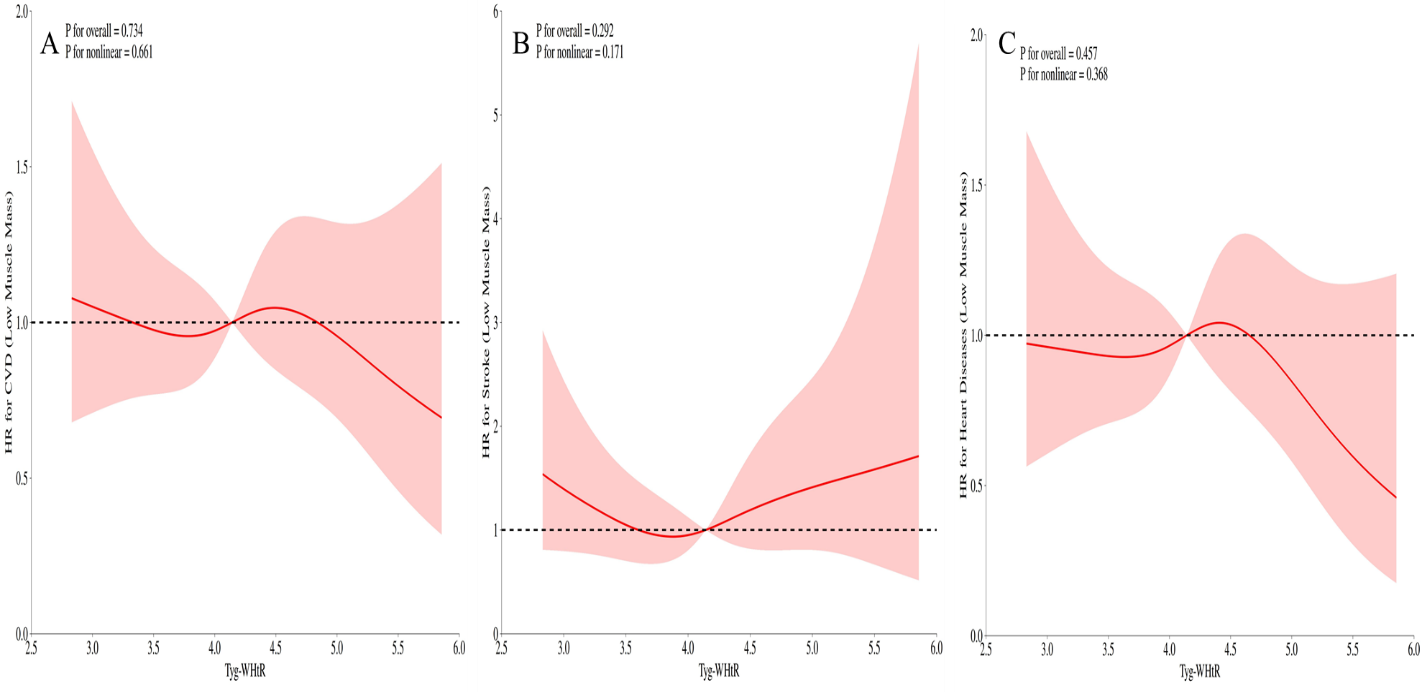
**

**Figure S1A–C** RCS curves depicting the association between the TyG-WHtR index and the risks of CVD, stroke, and heart diseases among participants with low muscle mass. The y-axis represents the HR (95% CI), whereas the x-axis displays TyG-WHtR index values. The model was adjusted for sex, age, education level, marital status, residence type, smoking status, alcohol consumption, hypertension, diabetes, SBP, DBP, TC, and CRP. The solid line and shaded area denote the estimated values and their corresponding 95% CIs, respectively.

**
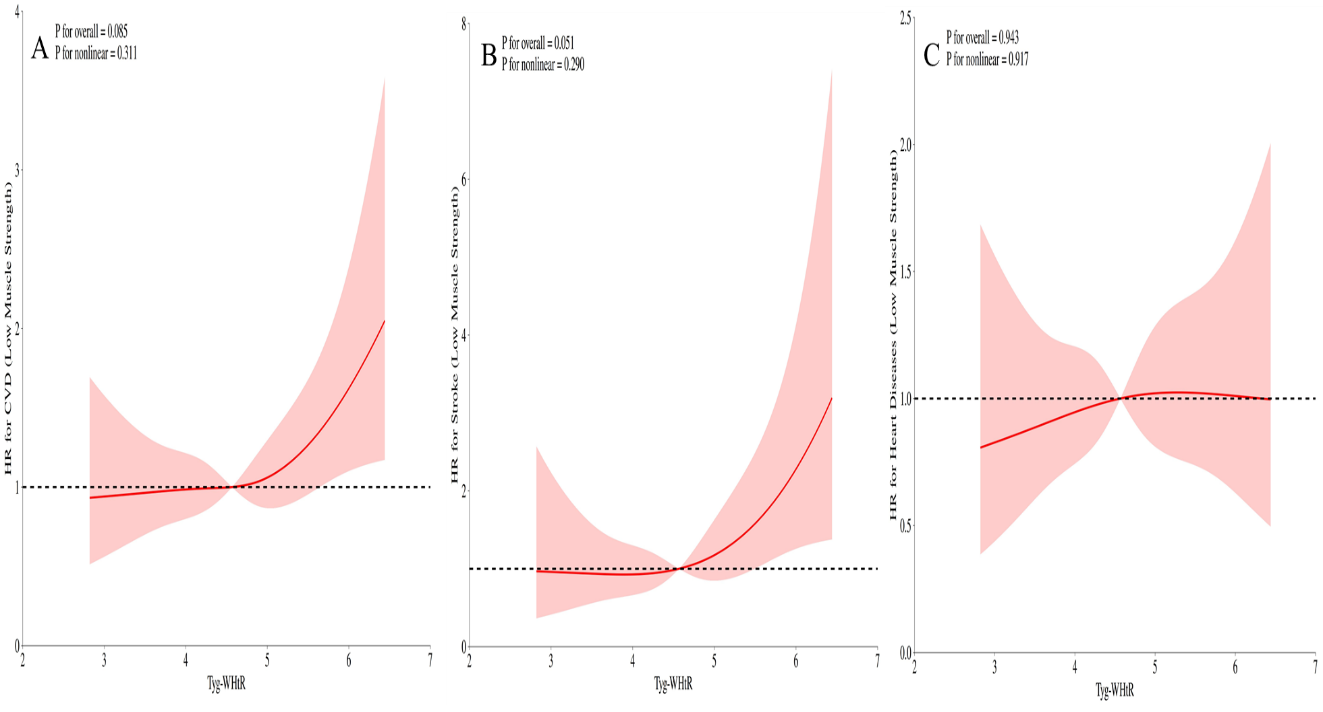
**

**Figure S2A–C** RCS curves illustrating the association between the TyG-WHtR index and the risks of CVD, stroke, and heart diseases among participants with low muscle strength. The y-axis represents the HR (95% CI), whereas the x-axis displays TyG-WHtR index values. The model was adjusted for sex, age, education level, marital status, residence type, smoking status, alcohol consumption, hypertension, diabetes, SBP, DBP, TC, and CRP. The solid line and shaded area denote the estimated values and their corresponding 95% CIs, respectively.

**
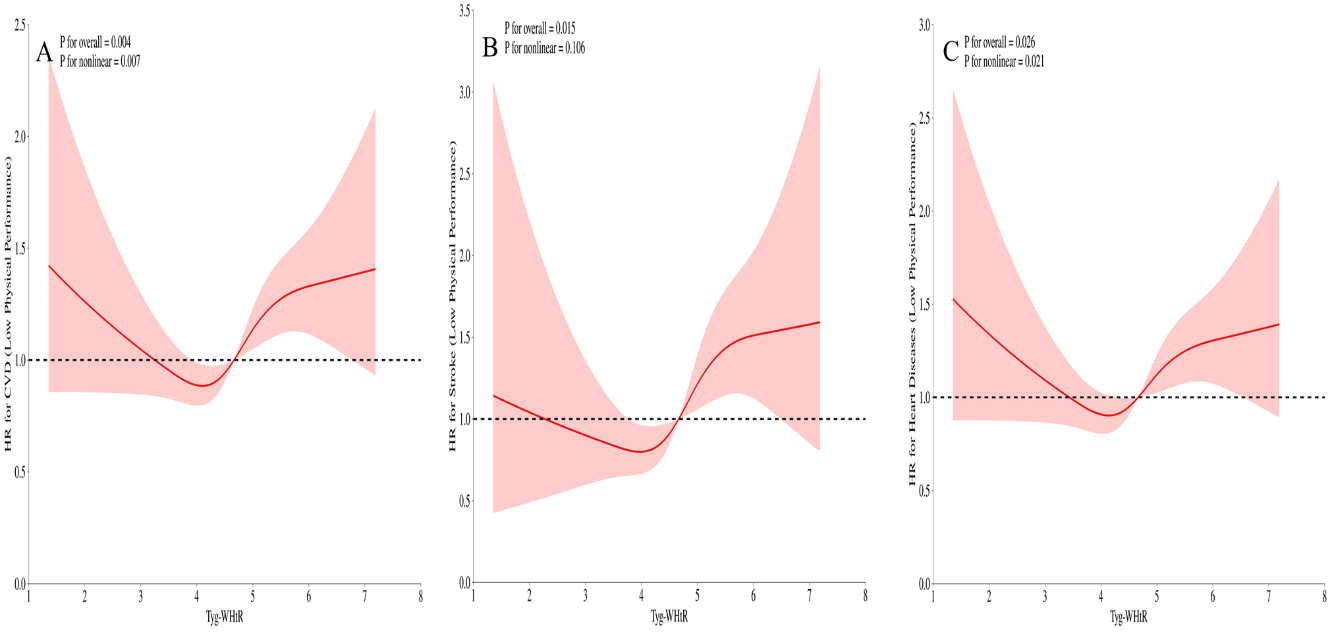
**

**Figure S3A–C** RCS curves illustrating the association between the TyG-WHtR index and the risks of CVD, stroke, and heart diseases among participants with low physical performance. The y-axis represents HR (95% CI), whereas the x-axis displays TyG-WHtR index values. The model was adjusted for sex, age, education level, marital status, residence type, smoking status, alcohol consumption, hypertension, diabetes, SBP, DBP, TC, and CRP. The solid line and shaded area denote the estimated values and their corresponding 95% CIs, respectively.
